# Prognostic value of cardiac magnetic resonance imaging parameters in left ventricular noncompaction with left ventricular dysfunction

**DOI:** 10.1186/s12872-022-02963-5

**Published:** 2022-12-06

**Authors:** Wei Bai, Rong Xu, Xiao Li, Huayan Xu, Hang Fu, Ruilai Hou, Ziqi Zhou, Wei Huang, Yining Wang, Yingkun Guo

**Affiliations:** 1 Department of Radiology, State Key Laboratory of Biotherapy, Key Laboratory of Obstetric & Gynecologic and Pediatric Diseases and Birth Defects of Ministry of Education, 20# South Renmin Road, Chengdu, 610041 Sichuan China; 2 Department of Radiology, Peking Union Medical College Hospital, Chinese Academy of Medical Sciences, Peking Union Medical College, No. 1 Shuaifuyuan, Dongcheng District, Beijing, 100730 China

**Keywords:** Cardiovascular magnetic resonance, Feature tracking, Late gadolinium enhancement, Left ventricular noncompaction, Prognosis

## Abstract

**Background:**

Cardiac magnetic resonance (CMR) has been used to diagnose and risk-stratify patients with left ventricular noncompaction (LVNC). The prognostic value of CMR parameters for LVNC, especially feature tracking (CMR-FT), is not well known in LVNC patients with left ventricular dysfunction. The present study aimed to investigate whether the combination of CMR-FT with traditional CMR parameters can increase the prognostic value of CMR for LVNC patients with reduced left ventricular ejection fraction (LVEF).

**Methods:**

A total of 123 candidates were retrospectively included in this multicenter study and 55 LVNC patients (mean age, 45.7 ± 16.2 years; 61.8% men) remained after applying the exclusion criteria. Clinical features, left ventricular (LV) function parameters, global and segment myocardial strain, and late gadolinium enhancement (LGE) were evaluated. The outcomes include the composite events of cardiovascular death, heart transplantation, hospitalization for heart failure, thromboembolic events, and ventricular arrhythmias.

**Results:**

After a median follow-up of 5.17 years (interquartile range: 0.17 to 10.58 years), 24 (36.8%) patients experienced at least one major adverse cardiovascular event (MACE). The myocardial strain parameters of patients with events were lower than those of patients without events. In the univariable Cox analysis, LVEF, the presence of LGE, global longitudinal strain (GLS) and segmental strains, including longitudinal strain at the apical level and radial and circumferential strain at the basal level, were significantly associated with MACEs. In the multivariate analysis, LGE (hazard ratio (HR) 3.452, 95% CI 1.133 to 10.518, *p* = 0.029) was a strong predictor of MACEs and significantly improved the predictive value (chi-square of the model after adding LGE: 7.51 vs. 13.47, *p* = 0.009). However, myocardial strain parameters were not statistically significant for the prediction of MACEs after adjusting for age, body mass index, LVEF and the presence of LGE and did not increase the prognostic value (chi-square of the model after adding GLS: 13.47 vs. 14.14, *p* = 0.411) in the multivariate model.

**Conclusions:**

The combination of CMR-FT with traditional CMR parameters may not increase the prognostic value of CMR in LVNC patients with reduced LVEF, while the presence of LGE was a strong independent predictor of MACEs and significantly improved the predictive value.

**Supplementary Information:**

The online version contains supplementary material available at 10.1186/s12872-022-02963-5.

## Background

Left ventricular noncompaction (LVNC) is a rare congenital disorder that is characterized by a bilayer myocardium with compacted and noncompacted layers, prominent trabeculations, and deep intertrabecular recesses connected with the left ventricular cavity [1]. LVNC is considered as a clinical phenotype with prognostic heterogeneity [2]. Myocardial dysfunction/heart failure is one of the major clinical manifestations and the main indicators of treatment and is largely associated with the outcomes of patients with LVNC [3–5]. However, predicting the prognosis of LVNC with left ventricular dysfunction is challenging and unclear. To guide individualized treatment strategies and monitoring in LVNC with left ventricular dysfunction, risk stratification tools are necessary.

Cardiac magnetic resonance (CMR) is the technique of choice for the diagnosis, and early detection of cardiomyopathy and the severity grading of LVNC [6]. Left ventricular dilation, left ventricular systolic dysfunction, and late gadolinium enhancement (LGE) have prognostic effects for patients with LVNC as evaluated using CMR [6–9]. With the emergence and development of new CMR imaging modalities, myocardial strain by CMR feature tracking (CMR-FT) has been proven to be a sensitive indicator for the assessment of abnormal cardiac deformation and plays an important role in the prediction of a series of heart diseases [10]. And CMR-FT significantly increases the diagnosis and prediction efficiency of adverse cardiovascular events when combined with the baseline clinical variables, left ventricular ejection fraction (LVEF) and LGE in a series of cardiomyopathies[11–13]. Although the studies on LVNC patients reported that the strain parameters were reduced even if LVEF was normal or supernormal, and the strain parameters are more sensitive to the changes in cardiac function [14–16], the prognostic value of myocardial strain by CMR is unknown in LVNC patients with left ventricular dysfunction.

The present study aimed to investigate whether the combination of CMR-FT with traditional MRI parameters can increase the prognostic value of MRI in an LVNC patient cohort with left ventricular dysfunction and to determine the optimal prediction model.

## Materials and methods

### Study population

This multicenter retrospective cohort study identified patients with the LVNC phenotype via a Boolean search of contrast-enhanced CMR reports between March 2009 and January 2020. Patients were queried in the CMR report database by searching the following keywords that describe LVNC: “left ventricular noncompaction”, “left ventricular hypertrabeculation”, and “spongiform cardiomyopathy”. The inclusion criteria were patients who fulfilled the following diagnostic criteria for LVNC: 1) bilayer myocardium with thin compacted and a thick noncompacted layers; 2) marked trabeculations with deep endomyocardial recesses; and 3) an end-diastolic noncompacted/compacted ratio > 2.3 in any LV segment in any long axis view [17]. The exclusion criteria were as follows: age < 18 years, incomplete or poor-quality CMR images, heart transplantation before CMR examination, any concurrent congenital or acquired heart disease, and normal left ventricular function (LVEF ≥ 55%).

**Fig. 1:**
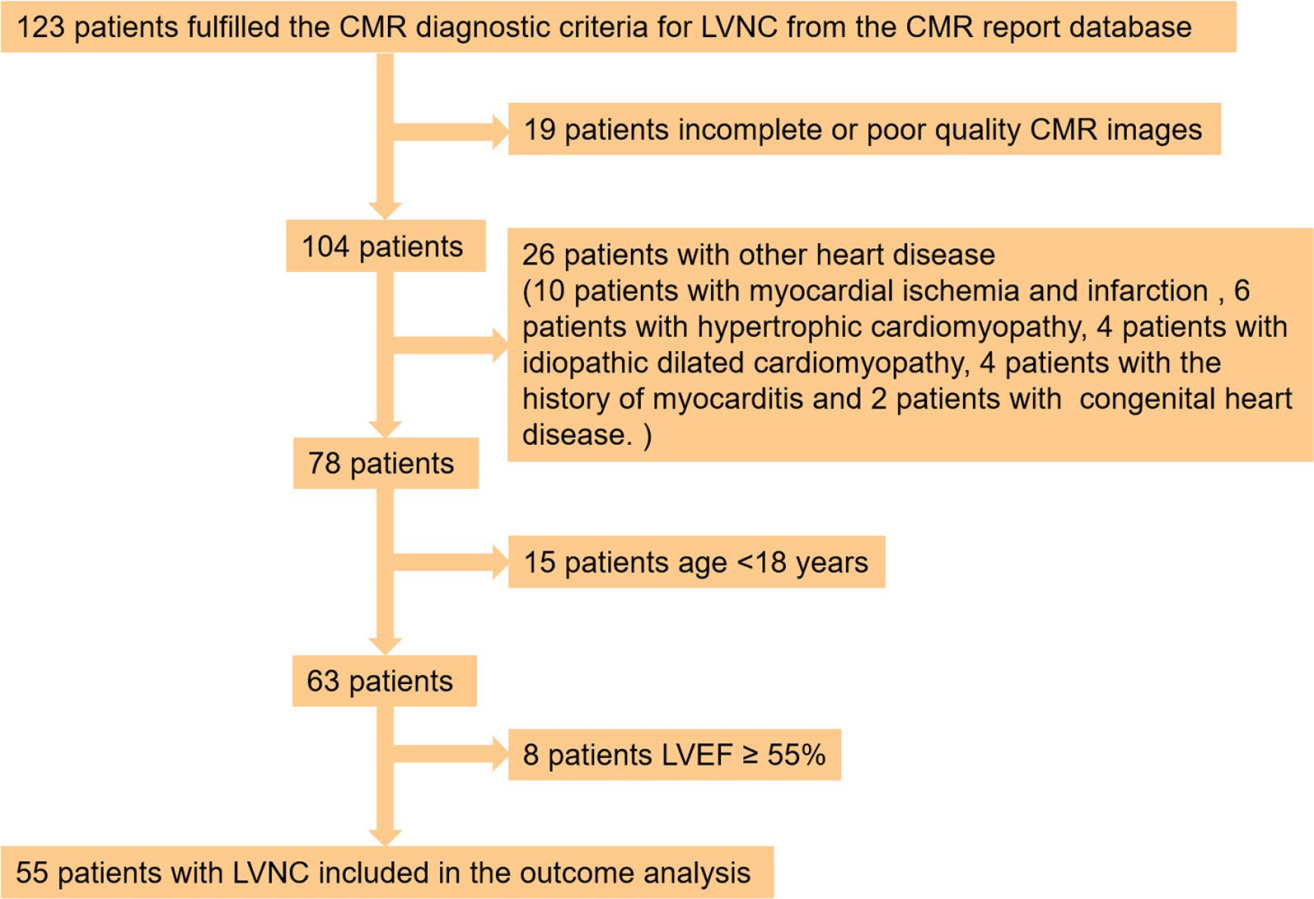
Flowchart describing the study cohort. *CMR* cardiovascular magnetic resonance; *LVNC* left ventricular noncompaction; *LVEF* left ventricular ejection fraction

A total of 123 candidates were identified from the CMR report database and 55 subjects remained after exclusion applying the exclusion criteria. The inclusion and exclusion criteria of the patients are summarized in Fig. 1. Clinical and CMR data were recorded in standard form at two centers by two experienced study physicians. In addition, we searched 51 CMR reports of healthy subjects as controls for segmental myocardial strain analysis, aged and sex matched to the LVNC patients. All data were made anonymous and analyzed at center A. The study protocol was approved by the Ethics Committee of the Peking Union Medical College Hospital, Sichuan University (K2019059) and West China Hospital of Sichuan University (756/2019), and written informed consent was obtained from all participants.

### CMR protocol

CMR images were obtained with the same type of scanner in two centers by using a 3.0-T whole body scanner (Skyra and Trio Tim; Siemens Medical Solutions, Erlangen, Germany). An 18-channel cardiac phased array coil and electrocardiogram triggering were used for CMR scanning. The adopted CMR protocol was the same in the two centers. Steady-state-free precession (SSFP) cine images were acquired for the assessment of left ventricular function and morphology before the contrast agent was injected, and they included two orientations, the short-axis view covering the full left ventricle from basis to apex and the 2-, 3- and 4-chamber long-axis views. LGE images for detecting myocardial scarring or fibrosis were obtained using a contrast-enhanced inversion recovery TrueFISP sequence for 10 ~ 15 min after an intravenous bolus of gadobenate dimeglumine (MultiHance 0.5 mmol/ml; Bracco, Milan, Italy). The dose of contrast agent was 0.1–0.2 ml/kg body weight at a flow rate of 2.5–3.0 ml/s and then injected with 20 ml of saline flush at a rate of 3.0 ml/s. The scan parameters of the two sequences were as follows: (1) SSFP sequence: slice thickness 6–8 mm, slice gap 0 mm, repetition time 3.42 ms, echo time 1.5 ms, flip angle 60°, and voxel size: 1.6 mm × 1.6 mm × 0.8 mm; (2) inversion recovery TrueFISP sequence: slice thickness 8 mm, repetition time 2.81 ms, echo time 1.04 ms and flip angle 55°.

### CMR image analysis

All collected images were transferred to the dedicated postprocessing software Cvi42 (Circle Cardiovascular Imaging, Calgary, Canada) and analyzed at center A by two expert radiologists blinded to patient baseline characteristics and radiological information. The left ventricular end-diastolic volume (EDV), left ventricular end-systolic volume (ESV), stroke volume (SV), and LVEF were calculated from the short-axis cine images in the short 3D module. EDV, ESV, and SV were corrected using BSA (LVEDVi, LVESVi, and LVSVi). Left ventricular epicardial and endocardial borders were manually traced in the 2-, 3-, and 4-chamber views of cine images at the end-diastole and the short-axis view at the end-diastole and end-systole by using the semiautomated feature tracking module. Global radial strain (GRS), global circumferential strain (GCS), and segmental radial and circumferential strain at the three levels, including apex, mid, and base, were calculated from the average of the peak strain of the short axis, and the global longitudinal strain (GLS) and segmental longitudinal strain at the three levels were calculated from the average of the peak strain of the long axis. Papillary muscles and noncompacted myocardium were excluded when the endocardial border was drawn. LGE extent was calculated as a percentage enhancement of myocardial mass from the LGE images. The presence of LGE was defined as myocardial enhancement with a signal intensity of > 5 SD above the mean signal intensity of the remote normal myocardium. The pattern and location of LGE were visually assessed by two experienced radiologists and classified as subendocardial, mid-wall, subepicardial, or transmural in distribution using standard American Heart Association 17 segment model.

### Follow-up

All patients were followed up on the telephone by using the standard questionnaire interview and the clinical medical records, electrocardiogram, ultrasound and the results of laboratory tests of all patients after CMR examination were queried, which was performed at center A by experienced physicians blinded to the CMR data. The clinical endpoints were major adverse cardiovascular events (MACEs), including cardiovascular death, heart transplantation, hospitalization for heart failure, thromboembolic events and ventricular arrhythmias defined as sustained or non-sustained ventricular tachycardia and ventricular fibrillation. The duration of follow-up was calculated from the date of the first CMR examination to the first occurrence of an endpoint.

### Statistical methods

Statistical analysis was performed using GraphPad Prism version 9.0 (GraphPad Software, La Jolla, California), IBM SPSS Statistics 23.0 software (IBM SPSS Statistics, IBM Corporation, Armonk, New York), and R software version 4.1.0 (R Foundation for Statistical Computing, Vienna, Austria). Continuous variables are presented as the mean ± standard deviation. Categorical variables are presented as absolute numbers (percentage). To compare the patient characteristics and CMR data in the groups, we compared continuous data by the Mann‒Whitney U test or independent Student’s t test and used chi-square analysis for categorial data. Correlations between LVEF and global strain parameters were assessed based on the Pearson correlation coefficient.

The cutoff value of the optimal receiver operating characteristic (ROC) curve for predicting MACEs was selected as the value for maximizing sensitivity and specificity. The survival rate was evaluated by Kaplan–Meier analysis. Univariate Cox regression analysis was used to identify potential clinical and CMR predictors of MACEs. Then these variables with *p* value ≤ 0.1 were included in multivariate Cox regression analysis to determine the independent predictors of the MACE. The hazard ratio (HR) and 95% confidence interval (CI) were calculated to estimate the risk associated with a particular variable. First, a baseline clinical Cox model building was performed as model 1. To investigate the incremental role, we sequentially added the traditional CMR markers, mainly including LVEF and the presence of LGE, to building model 2 and model 3. Finally, the parameters of CMR-FT (global and segmental myocardial strain) were added into model 3 to build other nested regression models. The -2Loglikelihood and c-index of each model for the prediction of MACEs were calculated. The likelihood ratio test and continuous net reclassification improvement (NRI) were used to evaluate the incremental prognostic value of these nested regression models. The intra- and interobserver reliability and agreement for continuous CMR variables were evaluated using the interclass correlation coefficients (ICCs) and Bland‒Altman analysis.

## Results

### Population characteristics

The baseline clinical characteristics for the controls and the patients with and without MACEs are shown in Table 1. A total of 24 (36.8%) patients experienced at least one MACE during a median follow-up of 5.17 years (interquartile range: 0.17 to 10.58 years). Four (16.7%) patients developed ventricular arrhythmias, five (20.8%) patients were hospitalized for heart failure, and 15 (62.5%) patients suffered from cardiac death. The causes of death included heart failure (n = 10), acute coronary syndrome (n = 2), malignant ventricular arrhythmias (n = 2) and infection after valve replacement (n = 1). The clinical characteristics were not significantly different between the two groups, except for age. Patients with MACEs were more likely to be older than those without MACEs (51.8 ± 17.1 years vs. 41.0 ± 13.9 years, *p* = 0.012).

**Table 1:** Clinical characteristics

	Controls (n = 51)	All patients (n = 55)	*p*-Valu*e*	Patients with events (n = 24)	Patients without events (n = 31)	*p*-Valu*e*
*Medical history*						
Age (years)	45.6 ± 15.1	45.7 ± 16.2	0.986	51.8 ± 17.1	41.0 ± 13.9	0.012*
Male (n, %)	27 (52.9)	34 (61.8)	0.422	18 (75)	16 (51.6)	0.077
BMI (kg/m2)	23.0 ± 1.9	23.9 ± 4.4	0.229	22.7 ± 4.5	25.3 ± 3.9	0.064
Heart rate (bpm)	74.0 ± 10.5	88.5 ± 24.2	0.001*	90.9 ± 29.2	86.2 ± 18.8	0.788
Systolic pressure (mmHg)	118.8 ± 11.0	115.9 ± 17.9	0.678	114.8 ± 17.3	117.0 ± 18.8	0.673
Diastolic pressure (mmHg)	81.6 ± 9.5	71.9 ± 13.6	0.074	71.1 ± 11.7	72.6 ± 15.2	0.719
Hypertension (n, %)	–	12 (21.8)	–	4 (16.7)	8 (25.8)	0.416
Smoking (n, %)	–	18 (32.7)	–	7 (29.2)	11 (35.5)	0.62
Diabetes (n, %)	–	8 (14.5)	–	5 (20.8)	3 (9.7)	0.245
Hypercholesterolemia (n, %)	–	16 (29.1)	–	7 (29.2)	9 (29.0)	0.991
Severe arrhythmia (n, %)	–	23 (41.8)	–	13 (54.2)	10 (32.3)	0.102
NYHA Class III/IV (n, %)	–	28 (50.9)	–	14 (58.3)	14 (45.2)	0.333
*Medical therapy*						
Beta-blocker (n, %)	–	36 (65.5)	–	15 (65.2)	21 (67.7)	0.685
Diuretics (n, %)	–	44 (80)	–	21 (87.5)	23 (74.2)	0.221
ACEI (n, %)	–	21 (38.2)	–	11 (45.8)	10 (32.3)	0.304
ABR (n, %)	–	13 (23.6)	–	4 (16.7)	9 (29)	0.284

Continuous data are shown as mean ± standard difference. Dichotomous data are shown as n (%)

^*^Means significant difference

*BMI* body mass index; *NYHA* New York Heart Association; *ACEI* angiotensin-converting enzyme inhibitors; *ARB* angiotensin receptor blocker

### CMR results

The CMR findings are shown in Table 2. Compared with the controls, the patients’ group had significantly lower LVEF, they had significantly larger EDVi and ESVi (*p* < 0.001). In patients’ group, the LVEF values were lower in patients with MACEs (22.9 ± 11.2% vs. 31.2 ± 13.7%, *p* = 0.025). All global and segmental strain parameters in patients’ group were significantly lower than in the controls (all *p* < 0.01). Compared with the patients without MACEs, global strain parameters were mainly attenuated in patients with MACEs, GRS (8.6 ± 5.6% vs. 13.3 ± 8.9%, *p* = 0.034), GCS (−7.4 ± 3.5% vs. −9.6 ± 4.8%, *p* = 0.091), and GLS (−4.7 ± 2.5% vs. −7.0 ± 3.2%, *p* = 0.004); Segmental strain parameters were also reduced, and the longitudinal strain at the apical level, radial and longitudinal strain at the mid level, and radial and circumferential strain at the basal level were significantly lower in patients with events (all *p* < 0.05). The decrease in global and segment strain at three different levels (apical, mid, and basal) in the same group are illustrated in Additional file 1: Fig. S1 in the appendix. The reduction in radial strain at the basal level, circumferential strain at the apical level, and longitudinal strain at the mid level were most pronounced, while the reduction in global strain was moderate.

**Table 2:** CMR parameters

	Controls (n = 51)	All patients (n = 55)	*p*-Value	Patients with events (n = 24)	Patients without events (n = 31)	*p*-Valu*e*
*Heart morphology*						
EDV (ml)	118.1 ± 26.3	259.6 ± 94.3	< 0.001*	274.6 ± 111.8	248.0 ± 78.2	0.378
EDVi (ml/m^2^)	65.8 ± 14.0	151.5 ± 56.2	< 0.001*	165.8 ± 65.6	140.4 ± 45.8	0.098
ESV (ml)	45.1 ± 11.9	193.0 ± 89.5	< 0.001*	212.5 ± 94.1	178.0 ± 84.1	0.158
ESVi (ml/m^2^)	25.1 ± 6.3	112.1 ± 51.9	< 0.001*	128.3 ± 54.7	99.5 ± 46.7	0.041*
SV (ml)	77.0 ± 18.1	66.6 ± 30.1	0.232	62.2 ± 37.2	70.0 ± 23.2	0.062
SVi (ml/m^2^)	40.7 ± 9.8	39.4 ± 19.8	0.705	37.5 ± 22.9	40.9 ± 17.2	0.169
LVEF (%)	62.9 ± 13.9	27.6 ± 13.2	< 0.001*	22.9 ± 11.2	31.2 ± 13.7	0.025*
*Peak strain (%)*						
Global						
Radial, GRS	33.0 ± 8.6	11.3 ± 7.9	< 0.001*	8.6 ± 5.6	13.3 ± 8.9	0.034*
Circumferential, GCS	−20.4 ± 2.3	−8.6 ± 4.4	< 0.001*	−7.4 ± 3.5	−9.6 ± 4.8	0.091
Longitudinal, GLS	−11.9 ± 3.8	−6.0 ± 3.1	< 0.001*	−4.7 ± 2.5	−7.0 ± 3.2	0.004*
Apical						
Radial, ARS	24.9 ± 12.9	9.8 ± 8.8	< 0.001*	8.5 ± 6.7	10.8 ± 10.1	0.497
Circumferential, ACS	−23.3 ± 3.4	−10.7 ± 6.2	< 0.001*	−9.5 ± 5.7	−11.6 ± 6.4	0.2
Longitudinal, ALS	−11.3 ± 8.3	−7.6 ± 4.1	0.005*	−5.8 ± 3.2	−9.0 ± 4.2	0.003*
Mid						
Radial, MRS	34.3 ± 10.1	10.6 ± 8.1	< 0.001*	8.3 ± 7.0	12.5 ± 8.5	0.031*
Circumferential, MCS	−20.5 ± 2.4	−8.3 ± 4.5	< 0.001*	−6.9 ± 3.6	−9.3 ± 4.9	0.079
Longitudinal, MLS	−13.2 ± 3.4	−5.7 ± 3.6	< 0.001*	−4.4 ± 3.3	−6.7 ± 3.6	0.011*
Basal						
Radial, BRS	46.7 ± 14.4	18.0 ± 11.6	< 0.001*	13.9 ± 8.3	21.2 ± 12.8	0.01*
Circumferential, BCS	−17.9 ± 2.3	−7.7 ± 3.3	< 0.001*	−6.7 ± 2.6	−8.5 ± 3.6	0.038*
Longitudinal, BLS	−11.7 ± 5.8	−5.2 ± 3.9	< 0.001*	−5.0 ± 3.0	−5.4 ± 4.6	0.41
LGE						
LGE extent (%)	–	9.7 ± 12.8	–	12.1 ± 10.6	8.0 ± 14.2	0.054
Patients with LGE (n, %)	–	34 (61.8)	–	19 (79.2)	15 (48.4)	0.02*
Subendocardial LGE (n, %)	–	84 (14.5)	–	53 (16.4)	31 (12.2)	0.156
Mid-wall LGE (n, %)	–	91 (15.7)	–	55 (17.0)	36 (14.1)	0.356
Subepicardial LGE (n, %)	–	16 (2.8)	–	11 (3.4)	5 (2.0)	0.321
Transmural LGE (n, %)	–	51 (8.8)	–	40 (12.4)	11 (4.3)	< 0.001*
Septum LGE (n, %)	–	62 (10.7)	–	36 (11.1)	26 (10.2)	0.787

Continuous data are shown as mean ± standard difference. Dichotomous data are shown as n (%)

*Means significant difference

*CMR* Cardiac magnetic resonance; *EDV* left ventricular end-diastolic volume; *ESV* left ventricular end-systolic volume; *SV* stroke volume; *LVEF* left ventricular ejection fraction; *GRS* global radial strain; *GCS* global circumferential strain; *GLS* global longitudinal strain; ARS, ACS, ALS, radial, circumferential and longitudinal peak strain at apical level; MRS, MCS, MLS, radial, circumferential and longitudinal peak strain at midlevel; BRS, BCS, BLS, radial, circumferential and longitudinal peak strain at basal level; *LGE* late gadolinium enhancement

The absolute value of global and segmental peak strain difference between controls and LVNC patients were provided in Additional file 1: Fig. S1

The presence of LGE (79.2% vs. 48.4%, *p* = 0.02) and the LGE extent (12.1 ± 10.6% vs. 8.0 ± 14.2%, *p* = 0.054) were higher in patients with MACEs. The most common pattern was subendocardial LGE and mid-wall LGE in all LVNC patients, the number of LGE segments was 84 (14.5%) and 91 (15.7%), respectively. The transmural LGE was more prevalent in patients with MACEs than in patients without MACEs (40 (12.4%) vs. 11 (4.3%), *p* < 0.001). However, no statistical significance was observed in the LGE extent between the two groups. The clinical characteristics and CMR parameters in LVNC patients with or without LGE are shown in Additional file 1: Table S1 in the appendix.

ROC analysis was performed for CMR parameters with significant differences between groups, and the results are displayed in Additional file 1: Table S2 in the appendix. The area under the curve of GRS, GLS, and LVEF was statistically significant (GRS: 0.669, GLS: 0.733, LVEF: 0.677, all *p* < 0.05). The cutoff values for GRS, GLS, and LVEF were ≤ 11.41%, > -6.23%, and ≤ 23.89%, respectively.

### Univariate analysis

In univariate Cox regression analysis (reported in Table 3), GLS (HR: 1.238; 95% CI: 1.018 to 1.506; *p* = 0.033) and LVEF (HR: 0.964; 95% CI: 0.929 to 0.999; *p* = 0.046) were univariate predictors of MACEs. Longitudinal strain at the apical level and radial and circumferential strain at the basal level were also univariate predictors of MACEs. The presence of LGE (HR: 2.768; 95% CI: 1.033 to 7.419; *p* = 0.043) was significantly associated with adverse events. However, the LGE extent (HR: 11.385; 95% CI: 0.441 to 293.9; *p* = 0.143) did not have prognostic value in the univariate Cox regression analysis.

**Table 3:** Univariable association with MACE

Clinical characters	HR (95% CI)	*p*-Valu*e*	CMR parameters	HR (95% CI)	*p*-Valu*e*	CMR parameters	HR (95% CI)	*p-*Valu*e*
Age	1.024 (0.998–1.050)	0.072	EDV	1.003 (0.998–1.008)	0.253	ARS	0.989 (0.938–1.044)	0.696
Sex	0.484 (0.192–1.222)	0.125	ESV	1.003 (0.999–1.008)	0.165	ACS	1.032 (0.961–1.108)	0.388
BMI	0.883 (0.788–0.988)	0.031*	SV	0.989 (0.971–1.008)	0.259	ALS	1.177 (1.024–1.353)	0.022*
Hypertension	0.440 (0.149–1.300)	0.137	LVEF	0.964 (0.929–0.999)	0.046*	MRS	0.947 (0.886–1.013)	0.113
Smoking	0.581 (0.238–1.419)	0.234	LGE extent	11.385 (0.441–293.9)	0.143	MCS	1.093 (0.981–1.219)	0.108
Diabetes	1.365 (0.504–3.696)	0.541	Patients with LGE	2.768 (1.033–7.419)	0.043*	MLS	1.083 (0.978–1.200)	0.125
Hypercholesterolemia	1.008 (0.385–2.637)	0.987	GRS	0.945 (0.881–1.014)	0.116	BRS	0.950 (0.901–1.002)	0.061
Severe arrhythmia	1.651 (0.723–3.771)	0.234	GCS	1.086 (0.976–1.210)	0.130	BCS	1.178 (1.014–1.369)	0.032*
NYHA Class III/IV	1.194 (0.507–2.816)	0.685	GLS	1.238 (1.018–1.506)	0.033*	BLS	1.000 (0.898–1.114)	0.997

*Means significant difference. The univariable HR and 95% CI are shown for the association with MACE

*MACE* major adverse cardiovascular event; *CI* confidence interval; *HR* hazard ratio

Other abbreviations as in Tables 1 and 2

### Multivariate analysis

**Fig. 2:**
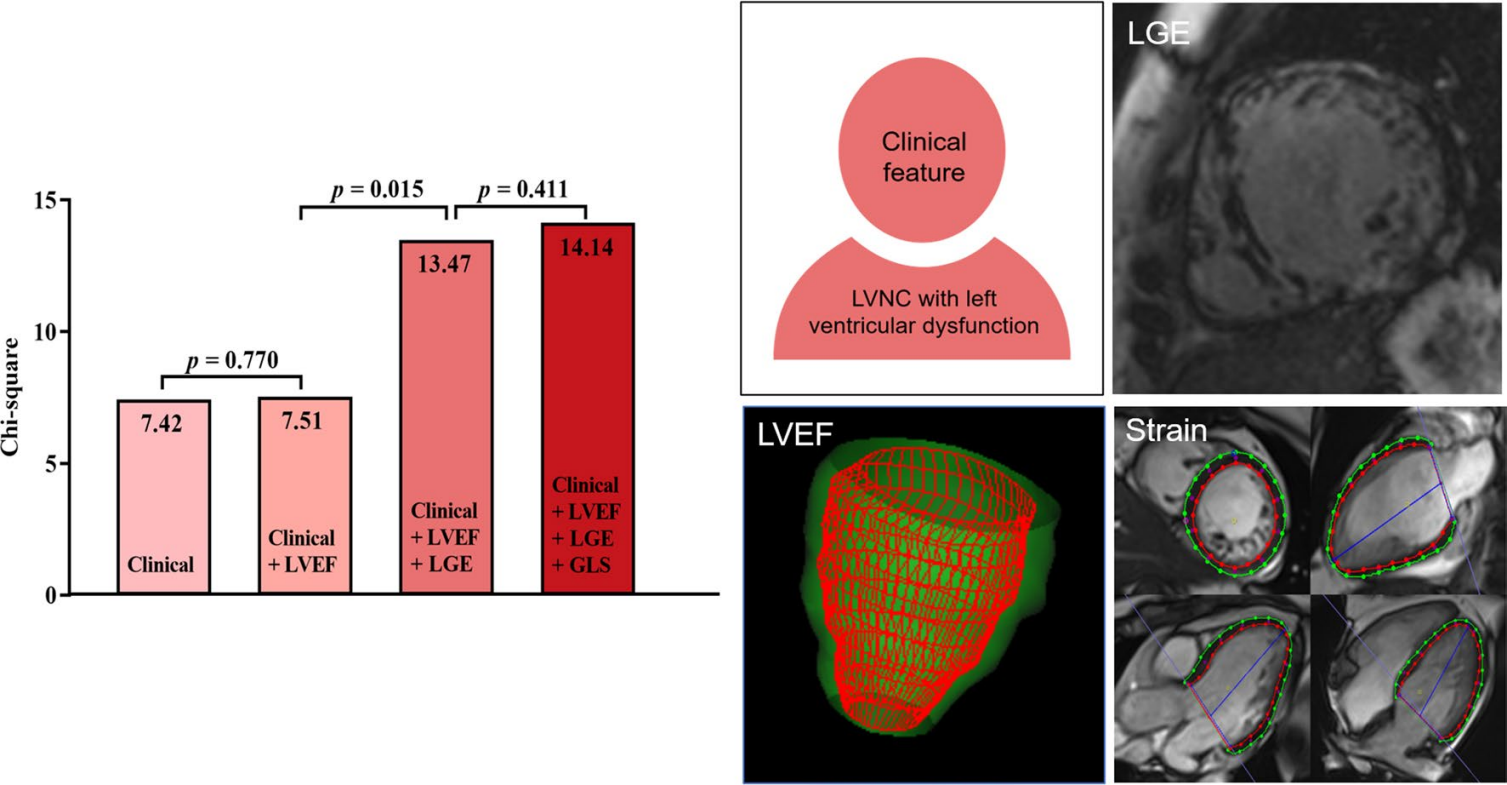
Incremental prognostic value of traditional CMR imaging markers and CMR-FT strain parameters. Nested multivariable survival models illustrate that feature tracking myocardial strain did not improve the prediction for major adverse cardiac event, and LGE was a robust independent predictor. *CMR* cardiovascular magnetic resonance; *LVEF* left ventricular ejection fraction; *GLS* global longitudinal strain; *LGE* late gadolinium enhancement; *FT* feature tracking

The different models by multivariate analysis are shown in Table 4. In multivariate analysis, which included age, body mass index (BMI), LVEF, the presence of LGE, and GLS (*p* < 0.1 in univariate Cox regression analysis), LGE was a strong and independent predictor of MACEs in model 3 and model 4. The presence of LGE (in model 3) significantly improved the model fit compared with model 2, including the clinical characteristics and LVEF (global chi-square test: 7.51 vs. 13.47, *p* = 0.015). Moreover, the continuous NRI increased significantly (model 2 vs. model 3: continuous NRI = 0.235, *p* = 0.049). In comparison with model 3, which includes the traditional CMR imaging markers of LVEF and the presence of LGE, the addition of GLS did not improve the model fit (global chi-square test: 13.47 vs. 14.14, *p* = 0.411) and GLS was no longer statistically significant for the prediction of MACEs in model 4. Figure 2 depicts the incremental values in predicting the MACEs by sequentially adding the traditional CMR imaging markers and CMR-FT strain parameter. The models including segmental strains by multivariate analysis are shown in Additional file 1: Table S3. The results showed that the addition of segmental strain parameters, longitudinal strain at the apical level and radial and circumferential strain at the basal level did not improve the model fit.

**Table 4:** Nested multivariable models for MACE

	Model 1		Model 2	
	Fit	*p*-Valu*e*	Fit	*p*-Valu*e*
–2Loglikelihood	120.698		120.604	
Likelihood ratio test	7.42	0.02	7.51	0.06
C-index	0.667 (0.532 to 0.802)		0.663 (0.524 to 0.802)	
Continuous NRI			0.082 (-0.363 to 0.441)	# vs. model 1: *p* = 0.878
*Covariates*				
Age	1.021 (0.993 to 1.049)	0.145	1.021 (0.993 to 1.049)	0.144
BMI	0.893 (0.796 to 1.003)	0.056	0.889 (0.787 to 1.003)	0.057
LVEF			1.006 (0.967 to 1.047)	0.757
Presence of LGE				
GLS				

	Model 3		Model 4	
	Fit	*p*-Valu*e*	Fit	*p*-Valu*e*
–2Loglikelihood	114.785		114.043	
Likelihood ratio test	13.47	0.009	14.14	0.01
C-index	0.712 (0.583 to 0.841)		0. 707 (0.570 to 0.844)	
Continuous NRI	0.235 (0.000 to 0.568)	#vs. model 2: *p* = 0.049	–0.226 (–0.527 to 0.306)	# vs. model 3: *p* = 0.585
*Covariates*				
Age	1.023 (0.993 to 1.053)	0.137	1.022 (0.993 to 1.053)	0.135
BMI	0.872 (0.776 to 0.978)	0.02	0.863 (0.767 to 0.970)	0.014
LVEF	1.017 (0.976 to 1.060)	0.425	1.006 (0.959 to 1.055)	0.816
Presence of LGE	3.452 (1.133 to 10.518)	0.029	3.241 (1.060 to 9.904)	0.039
GLS			0.882 (0.666 to 1.168)	0.381

The -2Loglikelihood and c-index (95%IC) of each model for the prediction of MACE were reported. The continuous NRI estimates (95% CI) for comparison of models were compared to evaluate the incremental prognostic value of these nested regression models. For the covariates, HR and 95% CIs were reported instead of fit statistics. The models including segment strains by multivariate analysis were showed in Additional file 1: Table S3. *NRI* net reclassification improvement. Other abbreviations as in Tables 1–3

### Incidence of MACEs and LGE

**Fig. 3:**
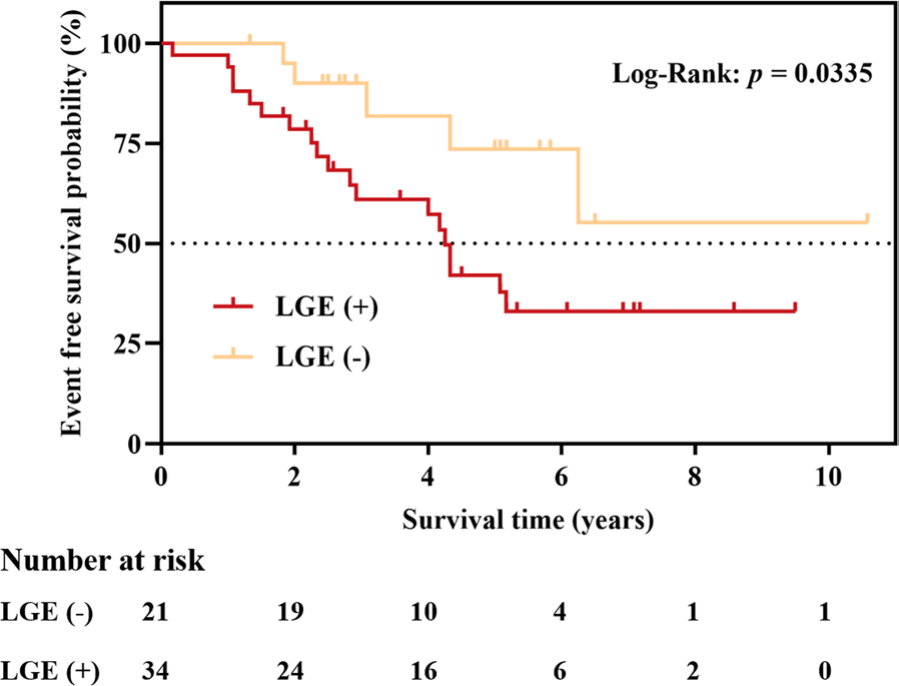
Kaplan–Meier analysis of prognosis according to LGE. Kaplan–Meier curve shows the time to cumulative survival and patients with LGE had poor outcomes. LGE, late gadolinium enhancement

The Kaplan‒Meier survival curve of the presence of LGE is displayed in Fig. 3 (log-rank = 4.522, *p* = 0.0335). The survival rates free from MACEs were 55.2% and 33.1% in patients without and with LGE, respectively. The median survival time was 4.25 years in patients with LGE.

### Myocardial strain and LVEF

**Fig. 4:**
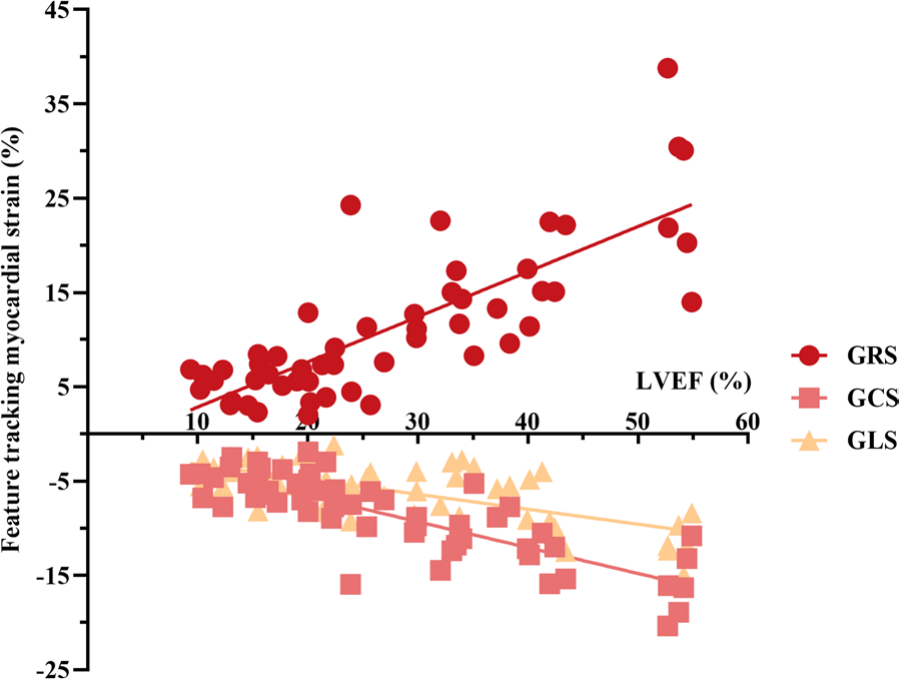
Correlation analysis of global myocardial strain by CMR-FT and LVEF. The global strain parameters were significantly correlated with LVEF. GRS: Pearson r = 0.7964, GCS: Pearson r = −0.8163, GLS: Pearson r = −0.6802, all *p* < 0.0001. CMR, cardiovascular magnetic resonance; FT, feature tracking; GRS, global radial strain; GCS, global circumferential strain; GLS, global longitudinal strain; LVEF, left ventricular ejection fraction

The strain parameters and LVEF were not independent predictors of MACEs in multivariate analysis. A strong correlation was observed between these parameters (correlation between LVEF and GRS: Pearson r = 0.7964; correlation between LVEF and GCS: Pearson r = −0.8163; correlation between LVEF and GLS: Pearson r = −0.6802; all *p* < 0.0001) (Fig. 4).

### Reproducibility analysis

A total of 20 patients were analyzed for intra- and inter-observer reliability and agreement. Additional file 1: Table S4 presents both intra- and interobserver variability for global and segmental myocardial strains. Consistency of measurements was good for all strain measurements and no significant differences were seen. With the exception of apical radial strain, mid radial strain and basal longitudinal strain, both intra- and interobserver ICC values (ICC = 0.770–0.993) were good to excellent for all analyzed strain measurements.

## Discussion

In the present study, we first evaluated the prognostic value of the combination of CMR-FT with traditional CMR markers, particularly LGE, in LVNC patients. The major findings are as follows: 1) the presence of LGE was a significant independent predictor of MACEs and can effectively improve the predictive ability of the risk prediction model in LVNC patients with reduced LV systolic function, 2) the global and segmental strain were lower in LVNC patients with MACEs than in those without MACEs, and 3) LV strain parameters from CMR-FT had no significant incremental prognostic value in these patients with reduced LV systolic function.

### Differential prognosis of LVNC according to LV strain

Strain parameters are the main noninvasive strategy for assessing systolic function of the left ventricle and are useful for the early detection and outcome evaluation of some heart diseases [18, 19]. Our results demonstrated that the global and segmental strain decreased in LVNC patients with MACEs. The same results were found in the pediatric population [20]. In univariate analysis, GLS was a predictor of MACEs. However, it was not an independent predictor of MACEs in multivariate analysis and could not increase the predictive value of the nested regression models in LVNC patients with reduced LVEF. However, recent publications highlight the predictive role of CMR-FT in cohorts of patients with the cardiovascular disease, especially GLS [11, 13, 21–23]. Myocardial deformation is an essential factor for maintaining normal global systolic function. Therefore, LV myocardial strain is associated with LVEF. Our results support this conclusion. The difference among these studies can mainly be attributed to the differences in the study cohort, in which all patients had myocardial dysfunction in our study. Compared with studies in which myocardial strain had incremental independent prognostic value, the LVEF of our subjects was generally lower (30.5% vs 55.9%, 46.4%, 44.9%) [12, 13, 24]. The study by Marciniak et al. [25] showed that when stroke volume was maintained, myocardial strain changed less and less as the left ventricular diameter increased. This may suggest that the sensitivity of myocardial strain gradually decreases with the decrease of decreasing LV function. Some studies have reported that myocardial strains and LVEF had no independent prognostic value in patients with dilated cardiomyopathy [26, 27]. In the present study, LVEF also failed to predict adverse events. Similar to these studies, the LVEF of the enrolled subjects was significantly reduced. The relationship between LVEF and survival probability was weaker when LVEF < 25% [28]. Therefore, LVEF may have superior predictive power in a cohort where most LVNC patients do not have severe left ventricular systolic dysfunction [29]. Based on the different results of the abovementioned studies, our findings are applicable to LVNC patients with reduced LV systolic function. Moreover, left ventricular function could be improved in patients who received medical therapy. Therefore, it may limit the application of global strain in predicting prognosis in LVNC patients with impaired systolic dysfunction.

In comparison with the global strain parameters, the segmental strain parameters can reveal the change in myocardial deformation in each segment. However, the inter- and interobserver reproducibility of some segmental strain values, such as apical radial strain, were not good and they should be used with caution within clinical studies. In the present study, the reduction of global strains was moderate compared with the three segmental strains. The reduction in circumferential and longitudinal strain at the mid and apical levels were more pronounced than that at the basal level. This result supported that myocardial noncompaction often occurs in the apical and middle segments [30, 31], and the range of myocardial circumferential and longitudinal motion increases gradually from the basal segment to the apical segment [32]. In the present study, the longitudinal strain at the apical level and radial and circumferential strains at the basal level were predictors of MACEs in univariate analysis. The addition of these segmental strains, especially the circumferential strain at the basal level, resulted in a higher c-index and chi-square value for the nested model compared with the addition of GLS, although they were not independent predictors. Some studies have shown that some segmental strain parameters can enhance the predictive performance in patients with cardiovascular disease [33, 34]. More research is required to explore the predictive value of segmental strains in LVNC patients.

### Prognostic importance of LGE for LVNC

LGE by cardiac MRI is a reference method for the noninvasive detection and quantification of myocardial fibrosis; moreover, it is a robust independent predictor of poor prognosis and is used extensively in various cardiomyopathy studies [35]. The results showed that the presence of LGE was a reliable predictor of MACEs, including hospitalization caused by heart failure, ventricular arrhythmias, and cardiovascular death. The patients with LGE had a 3.45-fold risk of MACEs than patients without LGE during a median follow-up of 5.17 years. Our results are consistent with the previous studies [7, 8, 36]. Moreover, the addition of the presence of LGE significantly improved the model fit compared with the model of age, BMI and LVEF based on a series of nested multivariable models.

Myocardial fibrosis can lead to changes in myocardial electrical properties. Wan J et al. showed that ventricular arrhythmias are common in patients with LGE [37]. In the present study, we found that the severe arrhythmia may occur in patients with LGE (50% vs. 28.6%). Nucifora et al. reported that myocardial fibrosis, qualitative and quantitative by LGE was observed in 55% patients with isolated LVNC and was correlated with clinical severity and ventricular dysfunction [6]. LVNC patients with LGE had low LVEF and high EDV, ESV, and NYHA functional class in our study. These results suggested that LVNC patients with LGE have lower cardiac function and severe clinical manifestations.

In the present study, the quantitative extent of LGE was considered, but no significant correlation was observed with clinical results. The discrepancy with some previous studies may be associated with the different study populations, small sample size, definitions of different outcomes, or the use of different statistical methods [6–8]. More studies with sufficient sample sizes are needed to further verify its prognostic value in the future.

### Study limitations

This study has some limitations. First, although this study was a multicenter study, the final patient cohort included in the study was limited. Uncontrollable confounders and selection bias may have been present. Second, most LVNC patients were treated with drugs because of cardiac dysfunction. However, we did not explore whether the different types and doses of drugs would affect the prognosis. Third, considering that the population of the study included the LVNC patients with reduced LVEF, the findings may not be universally applicable to all LVNC populations. In the future, we will further study the prognostic value of myocardial strain in patients with normal left ventricular function.

## Conclusions

This study further reinforces the predictive value of CMR-LGE in LVNC patients with reduced LVEF, while LV strain parameters from CMR-FT could not increase the prediction efficiency of the risk prognostic model in these patients.

## Supplementary Information

**Additional file 1 **: **Fig. S1.** Absolute value of global and segmental peak strain difference between controls and LVNC patients. **Table S1** Clinical characteristics and CMR parameters in LVNC patients with or without LGE. **Table S2** The ROC analysis for traditional CMR features and CMR feature tracking. **Table S3** Incremental value of the segmental strain. **Table S4** Intra- and inter-observer reproducibility for the myocardial strain parameters in LVNC patients.

## Data Availability

The datasets used and analyzed during the current study are available from the corresponding author on reasonable request.

## Abbreviations

LVNC: Left ventricular noncompaction
CMR: Cardiac magnetic resonance
LGE: Late gadolinium enhancement
CMR-FT: Cardiac magnetic resonance feature tracking
LVEF: Left ventricular ejection fraction
SSFP: Steady-state-free precession
EDV: End-diastolic volume
ESV: End-systolic volume
SV: Stroke volume
GRS: Global radial strain
GCS: Global circumferential strain
GLS: Global longitudinal strain
MACEs: Major adverse cardiovascular events
HR: Hazard ratio
CI: Confidence interval
NRI: Net reclassification improvement
ICC: Interclass correlation coefficients
BMI: body mass index
