# Supplementary material for: Prognostic value of cardiac magnetic resonance imaging parameters in left ventricular noncompaction with left ventricular dysfunction
Source: BMC Cardiovasc Disord. 2022 Dec 6;22:526. doi: 10.1186/s12872-022-02963-5 (PMC9724297; doi:10.1186/s12872-022-02963-5)
Supplement: Supplementary file 1 — Additional file 1 : Fig. S1. Absolute value of global and segmental peak strain difference between controls and LVNC patients. Table S1 Clinical characteristics and CMR parameters in LVNC patients with or without LGE. Table S2 The ROC analysis for traditional CMR features and CMR feature tracking. Table S3 Incremental value of the segmental strain. Table S4 Intra- and inter-observer reproducibility for the myocardial strain parameters in LVNC patients. [file 12872_2022_2963_MOESM1_ESM.docx]

**Appendix**

**
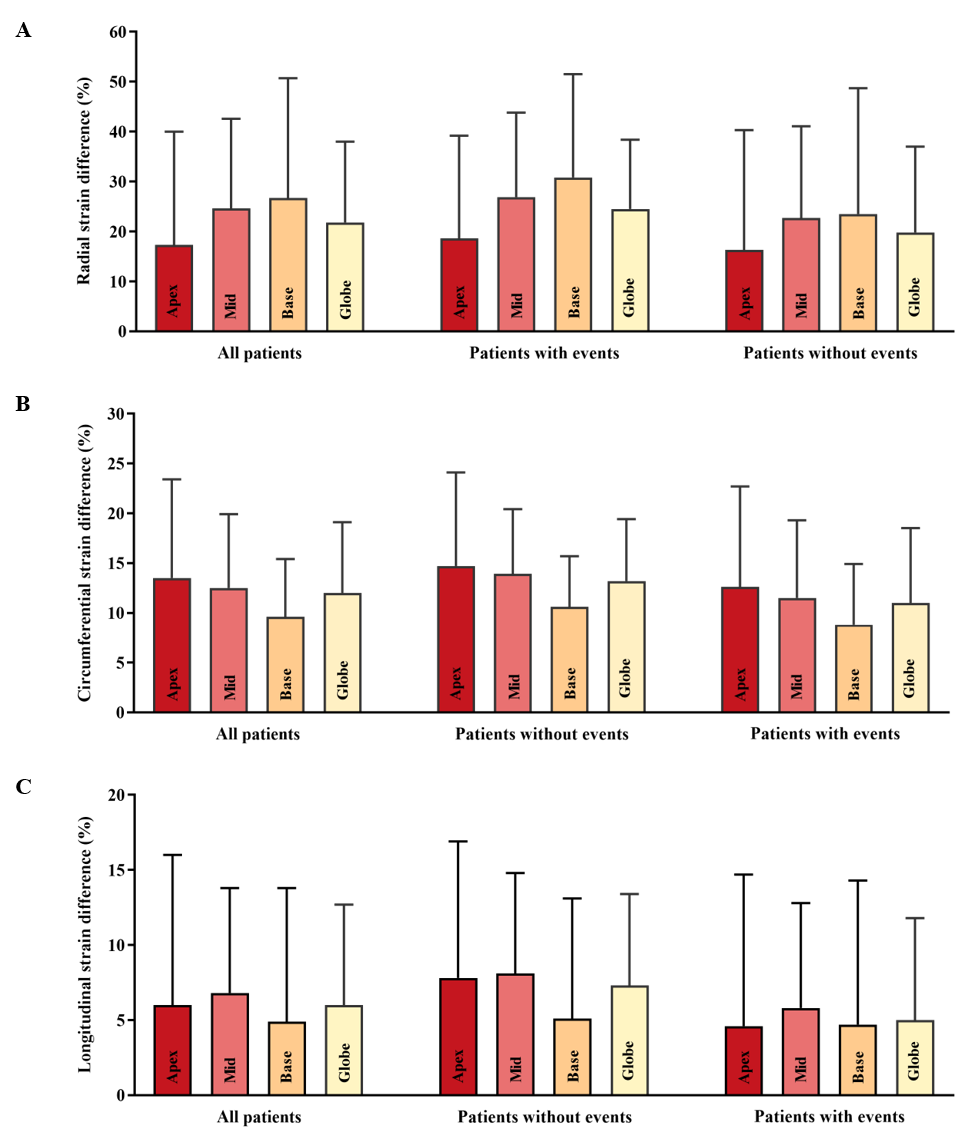
**

**Fig. S1.** Absolute value of global and segmental peak strain difference between controls and LVNC patients. We analyzed the myocardial strain of 51 healthy subjects as controls, aged and gender matched to the LVNC patients and calculated the difference of global and segmental strain between controls and LVNC patients. The reduction of radial strain at the basal level, circumferential strain and longitudinal strain at the mid and apical levels were pronounced, while the reduction of global strains was moderate.

**Table S1** Clinical characteristics and CMR parameters in LVNC patients with or without LGE.

|  | All patients  (n = 55) | Patients with LGE (n = 34) | Patients without LGE (n = 21) | p-Value |
| --- | --- | --- | --- | --- |
| Medical history | | | | |
| Age (years) | 45.7 ± 16.2 | 46.4 ± 15.7 | 44.5 ± 17.3 | 0.678 |
| Male (n, %) | 34 (61.8) | 24 (70.6) | 10 (47.6) | 0.088 |
| BMI (kg/m2) | 23.9 ± 4.4 | 24.4 ± 5.2 | 23.2 ± 2.7 | 0.429 |
| heart rate (bpm) | 88.5 ± 24.2 | 89.2 ± 26.4 | 87.3 ± 20.7 | 0.957 |
| systolic pressure (mmHg) | 115.9 ± 17.9 | 121.4 ± 18.2 | 106.9 ± 13.7 | 0.005* |
| diastolic pressure (mmHg) | 71.9 ± 13.6 | 75.1 ± 13.0 | 66.5 ± 13.0 | 0.032* |
| Hypertension (n, %) | 12 (21.8) | 9 (26.5) | 3 (14.3) | 0.288 |
| Smoking (n, %) | 18 (32.7) | 13 (38.2) | 5 (23.8) | 0.268 |
| Diabetes (n, %) | 8 (14.5) | 4 (11.8) | 4 (19) | 0.457 |
| Hypercholesterolemia (n, %) | 16 (29.1) | 10 (29.4) | 11 (28.6) | 0.947 |
| Severe arrhythmia (n, %) | 23 (41.8) | 17 (50) | 6 (28.6) | 0.118 |
| NYHA Class III/IV (n, %) | 28 (50.9) | 20 (58.8) | 8 (38.1) | 0.135 |
| Medical therapy | | | | |
| Beta-blocker (n, %) | 36 (65.5) | 22 (64.7) | 14 (66.7) | 0.882 |
| Diuretics (n, %) | 44 (80) | 27 (79.4) | 17 (81) | 0.890 |
| ACEI (n, %) | 21 (38.2) | 15 (44.1) | 6 (28.6) | 0.249 |
| ABR (n, %) | 13 (23.6) | 6 (17.6) | 7 (33.3) | 0.183 |
| CMR parameters | | | | |
| Heart morphology | | | | |
| EDV (ml) | 259.6 ± 94.3 | 277.8 ± 97.6 | 230.1 ± 82.6 | 0.069 |
| ESV (ml) | 193.0 ± 89.5 | 210.0 ± 88.6 | 165.6 ± 85.9 | 0.073 |
| SV (ml) | 66.6 ± 30.1 | 67.8 ± 31.8 | 64.6 ± 27.7 | 0.652 |
| LVEF (%) | 27.6 ± 13.2 | 26.3 ± 12.4 | 29.7 ± 14.4 | 0.446 |
| Peak strain (%) | | | | |
| Radial, GRS | 11.3 ± 7.9 | 9.9 ± 6.4 | 13.4 ± 9.7 | 0.260 |
| Circumferential, GCS | -8.6 ± 4.4 | -7.8 ± 3.8 | -10.0 ± 5.1 | 0.141 |
| Longitudinal, GLS | -6.0 ± 3.1 | -5.8 ± 3.1 | -6.4 ± 3.1 | 0.368 |

Continuous data are shown as mean±standard difference. Dichotomous data are shown as n (%). * means significant difference.

CMR, cardiac magnetic resonance; LVNC, left ventricular noncompaction; LGE, late gadolinium enhancement; BMI, body mass index; NYHA, New York Heart Association; ACEI, angiotensin-converting enzyme inhibitors; ARB, angiotensin receptor blocker; EDV, left ventricular end-diastolic volume; ESV, left ventricular end-systolic volume; SV, stroke volume; LVEF, left ventricular ejection fraction; GRS, global radial strain; GCS, global circumferential strain; GLS, global longitudinal strain.

**Table S2** The ROC analysis for traditional CMR features and CMR feature tracking.

| CMR parameters | Area under the ROC curve | | | |  | Youden’s index | | | |
| --- | --- | --- | --- | --- | --- | --- | --- | --- | --- |
|  | AUC | SD | 95%IC | p-Value |  | Youden’s index | Cut-off | Sensitivity (%) | Specificity (%) |
| Traditional CMR features | | | | | | | | | |
| LVEF | 0.677 | 0.0732 | 0.538 to 0.797 | 0.0153* |  | 0.2796 | ≤23.89 | 66.67 | 61.29 |
| CMR feature tracking | | | | | | | | | |
| GRS | 0.668 | 0.074 | 0.528 to 0.789 | 0.0231* |  | 0.3495 | ≤11.41 | 83.33 | 51.61 |
| GLS | 0.733 | 0.0687 | 0.597 to 0.843 | 0.0007* |  | 0.3817 | >-6.23 | 83.33 | 54.84 |
| ALS | 0.733 | 0.0697 | 0.597 to 0.843 | 0.0008* |  | 0.4449 | >-4.79 | 54.17 | 90.32 |
| MRS | 0.671 | 0.0748 | 0.531 to 0.791 | 0.0225* |  | 0.3347 | ≤6.4 | 62.5 | 70.97 |
| MLS | 0.702 | 0.0736 | 0.564 to 0.818 | 0.006* |  | 0.4409 | >-5.1 | 66.67 | 77.42 |
| BRS | 0.703 | 0.0738 | 0.565 to 0.819 | 0.006* |  | 0.4556 | ≤18.61 | 87.5 | 58.06 |
| BCS | 0.664 | 0.0751 | 0.524 to 0.786 | 0.029* |  | 0.3710 | >-5.52 | 50 | 87.10 |

* means significant difference.

ROC, receiver operating characteristic; AUC, area under the curve; CI, confidence interval. ARS, ACS, ALS, radial, circumferential and longitudinal peak strain at the apical level; MRS, MCS, MLS, radial, circumferential and longitudinal peak strain at the mid level; BRS, BCS, BLS, radial, circumferential and longitudinal peak strain at the basal level; Other abbreviations as in Table S1.

**Table S3** Incremental value of the segmental strain.

| **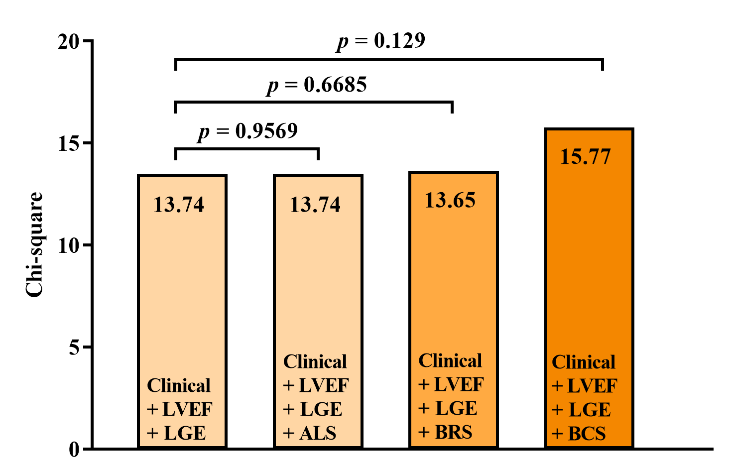** | | | | | | | | |
| --- | --- | --- | --- | --- | --- | --- | --- | --- |
|  | Apical longitudinal strain | |  | Basal radial strain | |  | Basal circumferential strain | |
|  | Fit | p-Value |  | Fit | p-Value |  | Fit | p-Value |
| -2Loglikelihood | 114.771 |  |  | 114.586 |  |  | 112.464 |  |
| Likelihood ratio test | 13.47 | 0.02 |  | 13.65 | 0.02 |  | 15.77 | 0.008 |
| C-index | 0.712 (0.583 to 0.841) |  |  | 0.721 (0.590 to 0.852) |  |  | 0.747 (0.606 to 0.880) |  |
| Continuous NRI | 0.298 (-0.167 to 0.516) | vs. model 3: *p* = 0.269 |  | 0.049 (-0.423 to 0.441) | vs. model 3: *p* = 0.915 |  | 0.174 (-0.252 to 0.480) | vs. model 3: *p* = 0.428 |
| Covariates | | | | | | | | |
| Age | 1.023 (0.992 to 1.055) | 0.142 |  | 1.023 (0.993 to 1.054) | 0.131 |  | 1.026 (0.994 to 1.059) | 0.115 |
| BMI | 0.871 (0.776 to 0.978) | 0.019 |  | 0.866 (0.768 to 0.976) | 0.019 |  | 0.839 (0.738 to 0.953) | 0.007 |
| LVEF | 1.016 (0.969 to 1.065) | 0.519 |  | 1.027 (0.966 to 1.092) | 0.387 |  | 1.055 (0.992 to 1.121) | 0.088 |
| Presence of LGE | 3.449 (1.132 to 10.512) | 0.020 |  | 3.485 (1.138 to 10.673) | 0.029 |  | 3.596 (1.145 to 11.293) | 0.028 |
| Segment strain | 0.989 (0.824 to 1.188) | 0.908 |  | 0.978 (0.886 to 1.079) | 0.659 |  | 1.252 (0.935 to 1.677) | 0.131 |

NRI, net reclassification improvement. Abbreviations as in Table S1.

**Table S4** Intra- and inter-observer reproducibility for the myocardial strain parameters in LVNC patients (n=20).

|  | Variability | Mean bias ± SD | p-Value | Limits of agreement | ICC (95% CI) |
| --- | --- | --- | --- | --- | --- |
| BRS | Intra-observer | 2.277 ± 8.932 | 0.3159 | -15.23 to 19.78 | 0.851 (0.662 to 0.938) |
|  | Interobserver | 1.191 ± 4.994 | 0.3390 | -8.597 to 10.98 | 0.937 (0.848 to 0.974) |
| BCS | Intra-observer | -0.2906 ± 1.133 | 0.2867 | -2.511 to 1.930 | 0.975 (0.938 to 0.990) |
|  | Interobserver | -0.0740 ± 1.912 | 0.7048 | -3.822 to 3.674 | 0.922 (0.815 to 0.969) |
| BLS | Intra-observer | -0.6685 ± 2.799 | 0.4411 | -6.154 to 4.817 | 0.681 (0.361 to 0.859) |
|  | Interobserver | 0.4150 ± 2.518 | 0.4710 | -4.520 to 5.350 | 0.770 (0.508 to 0.902) |
| MRS | Intra-observer | -0.1275 ± 4.627 | 0.6950 | -9.196 to 8.941 | 0.899(0.762 to 0.959) |
|  | Interobserver | 0.4385 ± 7.596 | 0.7069 | -14.45 to 15.33 | 0.717 (0.408 to 0.878) |
| MCS | Intra-observer | -0.0110 ± 0.7395 | 0.7939 | -1.460 to 1.438 | 0.993 (0.983 to 0.997) |
|  | Interobserver | -0.3295 ± 1.180 | 0.5194 | -2.643 to 1.984 | 0.980 (0.951 to 0.992) |
| MLS | Intra-observer | -0.2045 ± 1.556 | 0.8891 | -3.255 to 2.846 | 0.912 (0.793 to 0.964) |
|  | Interobserver | -0.0750 ± 0.9972 | 0.7657 | -2.029 to 1.879 | 0.961 (0.905 to 0.984) |
| ARS | Intra-observer | 1.135 ± 9.484 | 0.9130 | -17.45 to 19.72 | 0.643 (0.290 to 0.842) |
|  | Interobserver | 0.0350 ± 8.228 | 0.9595 | -16.09 to 16.16 | 0.574 (0.178 to 0.808) |
| ACS | Intra-observer | -0.1640 ± 3.905 | 0.2985 | -7.818 to 7.490 | 0.881 (0.724 to 0.951) |
|  | Interobserver | -0.8760 ± 4.456 | 0.3442 | -7.857 to 9.609 | 0.836 (0.636 to 0.931) |
| ALS | Intra-observer | -0.1850 ± 1.226 | 0.1728 | -2.588 to 2.218 | 0.968 (0.923 to 0.987) |
|  | Interobserver | -1.154 ± 3.143 | 0.1883 | -7.314 to 5.007 | 0.810 (0.582 to 0.920) |
| GRS | Intra-observer | -1.198 ± 3.064 | 0.1918 | -7.202 to 4.807 | 0.94 (0.85 to 0.976) |
|  | Interobserver | 0.1605 ± 5.256 | 0.7326 | -10.14 to 10.46 | 0.842 (0.643 to 0.935) |
| GCS | Intra-observer | 0.102 ± 1.672 | 0.3453 | -3.175 to 3.379 | 0.967 (0.919 to 0.987) |
|  | Interobserver | 0.312 ± 2.711 | 0.3450 | -5.002 to 5.626 | 0.903 (0.773 to 0.96) |
| GLS | Intra-observer | 0.079 ± 1.688 | 0.8518 | -3.229 to 3.387 | 0.904 (0.774 to 0.961) |
|  | Interobserver | -0.1515 ± 1.495 | 0.7190 | -3.082 to 2.779 | 0.921 (0.812 to 0.968) |

ICC, intra-class coefficient; CI, confidence interval. Other abbreviations as in Table S1.
